# Acute Onset of Psychosis in a Patient with a Left Temporal Lobe Arachnoid Cyst

**DOI:** 10.1155/2014/204025

**Published:** 2014-02-11

**Authors:** Alexander Mironov, Sabu John, Jonathan Auerbach, Ghassan Jamaleddine

**Affiliations:** ^1^Department of Medicine, SUNY Downstate Medical Center, Brooklyn, NY 11203, USA; ^2^Department of Medicine, Kings County Hospital Center, Brooklyn, NY 11203, USA

## Abstract

Arachnoid cysts are considered a rare neurological tumor, few of which exhibit any symptomatology. A 38-year-old Haitian American female with no past psychiatric history presented with rapid onset of psychosis. Workup for medical etiology proved to be within normal limits, with the exception of a left temporal lobe arachnoid cyst. The purpose of this paper is to add to a number of existing case reports that suggest a relationship between such lesions and psychiatric illness.

## 1. Introduction

Arachnoid cysts are considered to be a relatively rare neurological tumor, accounting for roughly 1% of intracranial space occupying lesions [1]. Of patients undergoing a brain MRI, only 1.4% are identified to have an arachnoid cyst. Most of these (34%) are found to be in the mid cranial fossa, the majority (70%) being left sided. Even more rare are patients who become symptomatic (5%); among the most common complaints are headache, ataxia, seizures, dizziness, and visual changes [2].

Numerous articles have also reported patients with intracranial arachnoid cysts presenting with psychiatric illness as their main symptom [3–5]. Although no clear-cut mechanism has yet been identified, there are copious instances of patients being cured of their psychiatric symptoms following neurosurgical intervention [6–9]. Meanwhile, the remainder of patients were managed medically.

We would like to present a case in which a middle aged female develops rapid onset psychosis and delirium. After extensive medical workup for the etiology of her condition, there were no abnormalities to be found, with the exception of a left temporal lobe arachnoid cyst.

## 2. Case Presentation

Mrs. G is a 38-year-old Haitian American female brought into the emergency department by her family due to a sudden onset of psychotic behavior and delirium. She has no past psychiatric history and her unusual behavior started roughly one week prior to admission. The patient takes no home medications and has no family history of psychiatric illness. She works as a nurse in a nearby hospital, takes care of two children, and lives in the same apartment building as her mother, who was the original witness to the patient's change in behavior. Her symptoms included religious and persecutory delusions as well as delusions of control. Also, she has had zero to little sleep over the past week. The patient was alert and oriented to person, place, and time. She remained cooperative during her interview, however she became irritable upon questioning of her delusions. Her thought process was linear, logical, and goal directed. Judgment was intact, except when related to her delusions. Furthermore, she portrayed poor insight into her medical condition, symptoms, and need for treatment. The patient was placed on 1 : 1 observation as a precaution due to a desire to escape to home, deemed incapacitated to leave against medical advice, and started on risperidone 0.5 mg PO BID. Additionally, she received haloperidol 5 mg and lorazepam 2 mg IM PRN multiple times throughout her admission for agitation control.

**Figure 1:**
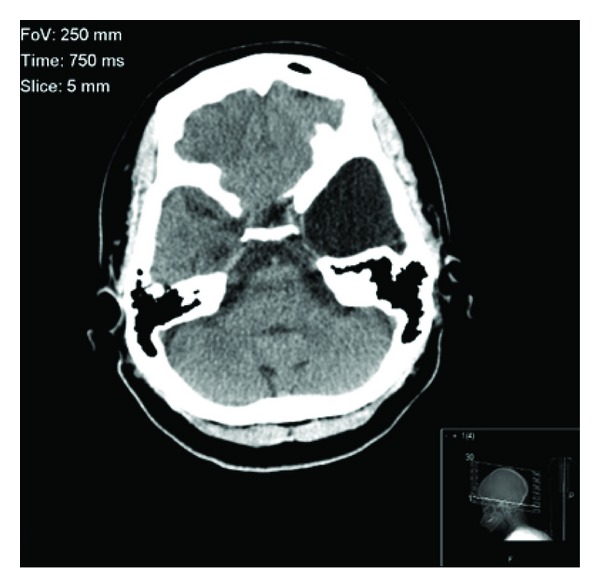
Cranial CT without contrast in the horizontal plane, showing an arachnoid cyst in the region of the left temporal lobe.

**Figure 2:**
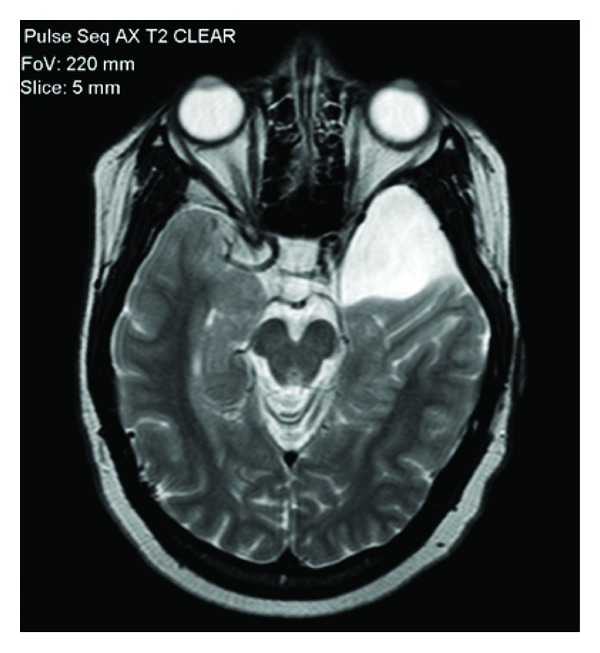
Cranial MRI without contrast in the horizontal plane, demonstrating the mass effect on the left temporal lobe by the arachnoid cyst.

After the initial psychiatric consultation, she was admitted to the medicine service to rule out any medical etiology of her psychiatric symptoms. The medical workup included screening for electrolyte abnormalities, urine toxicology, ethyl alcohol, cannabinoids, acetaminophen, salicylate, serum glucose, thyroid function, cobalamin, folate, rapid plasma reagin, and borrelia burgdorferi, all of which came back within normal limits. Neurology consultation revealed no focal neurological deficits and recommended electroencephalography (EEG) and lumbar puncture (LP). EEG showed no signs of seizure activity. However, there was intermittent polymorphic slowing over both temporal regions, which is indicative of mild cerebral dysfunction. Cerebrospinal fluid analysis obtained from LP was within normal limits, negative for bacterial antigen agglutination, and cultures had no growth. Lastly, a CT and MRI of the head revealed a cystic lesion within the anterior aspect of the left middle cranial fossa which demonstrated mass effect on the left temporal lobe and was compatible with an arachnoid cyst. The lesion measured 3.7 × 3.1 × 3 cm (see Figures 1 and 2).

Neurological surgery was promptly consulted; however, due to the lack of neurological symptoms; the surgeons recommended against any neurosurgical intervention and opted for medical management.

After four days of hospitalization and an extensive workup, a clear etiology for Mrs. G's rapid onset delirium and psychosis was not identified. Nevertheless, she showed marked improvement with her medication regimen. Prior to discharge, the patient's delusions could not be elicited, there was improved insight, and she was no longer a danger to herself and others. Still, she was refusing further workup of her cyst and did not accept the suggestion that it may be related to her psychiatric symptoms. Arrangements were made for both neurologic and psychiatric follow-up as an outpatient.

## 3. Discussion

This patient's clinical presentation is a characteristic of a rapid onset psychotic disorder accompanied by delirium. Following thorough evaluation, medical causes for her presentation were ruled out, except for a left temporal lobe arachnoid cyst being the only aberrant finding. A review of the literature revealed numerous cases in which patients with psychiatric symptoms are discovered to have an arachnoid cyst.

More specifically, there has been evidence to suggest a link between structural abnormalities of the left temporal lobe and schizophrenia [10]. Patients undergoing right temporal lobectomy for chronic localized epilepsy have also been shown to develop new onset psychiatric illness following the procedure [11]. In addition to schizophrenia [6, 10] and schizophreniform disorder [8], a multitude of other symptoms have been reported. Auditory hallucinations, delusions of persecution [3, 4, 9], delusions of hypochondria [4], and conversion disorder [6] have all been documented in association with arachnoid cysts. Aggressive behavior [4] and uncharacteristic violent behavior [12] have also been noted.

Patients who experienced a resolution of symptoms following neurosurgical intervention provide evidence that arachnoid cysts may be the underlying etiology to their psychiatric manifestations. Surgical techniques used include craniotomy alone [6], excision [6, 8], and drainage [9]. In another incidence, a patient presenting with depression and schizophrenia-like symptoms was found to have an intraventricular ependymal cyst. The lesion was resected through craniectomy and endoscopy, with subsequent remission of symptoms [7].

Despite the reported success of neurological surgery, it is still considered a very high risk treatment option with no uniform and established guidelines. Progressive hydrocephaly and intracranial hypertension seem to be the only situations mandating surgery [1]. Also, the majority (96.8%) of arachnoid cysts show no change in size over a mean of 3.8-year follow-up period [2]. Hence, conservative medical management seems to be the preferred approach for the majority of cases [1].

In the case we present, the patient was discharged in stable condition on risperidone 0.5 mg PO BID and given follow-up instructions. Four months later, she presented to our psychiatric emergency room with a relapse of psychosis attributed to medication nonadherence for an unspecified time period. Her symptoms fully resolved after restarting risperidone 1 mg PO QPM. Arrangements were again made for outpatient follow-up, this time involving the patient's mother with the supervision of treatment.

## 4. Conclusion

The association between arachnoid cysts and psychiatric illness remains a controversial and frequently overlooked phenomenon. Despite this, there are a growing number of case reports that suggest this combination of findings is not simply a coincidence. For this reason, it may be worthwhile to take cranial imaging into consideration when establishing a diagnosis and treatment plan. Rapid resolution of symptoms following neurosurgical intervention perhaps provides the most convincing evidence of a linkage between the two. Further studies are warranted to provide better evidence and confirmation of this relationship.
